# Scaffolding theory of maturation, cognition, motor performance, and motor skill acquisition: a revised and comprehensive framework for understanding motor–cognitive interactions across the lifespan

**DOI:** 10.3389/fnhum.2025.1631958

**Published:** 2025-08-12

**Authors:** Thomas Jürgen Klotzbier, Nadja Schott

**Affiliations:** Department of Sport and Exercise Science, University of Stuttgart, Stuttgart, Germany

**Keywords:** scaffolding theory, neuroplasticity, motor–cognitive interaction, fitness, motor learning, autonomy and motivation in learning, cognitive enhancement, dual task

## Abstract

The Scaffolding Theory of Maturation, Cognition, Motor Performance, and Motor Skill Acquisition (SMART COMPASS) provides a revised, integrative framework for understanding the dynamic relationship between motor and cognitive systems across the lifespan. Integrating concepts from the Scaffolding Theory of Aging, the Integrated Framework for Cognitive and Motor Skill Development, and the OPTIMAL Theory of Motor Learning, the model demonstrates how neural, environmental, and behavioral factors jointly shape cognitive and motor performance. Its unique contribution lies in bridging neurobiological mechanisms (e.g., neuroplasticity and cognitive reserve), psychological drivers (e.g., autonomy and self-efficacy), and motor learning principles into a unified, lifespan-oriented approach. Unlike existing frameworks, SMART COMPASS explicitly links structured physical training and motor skill learning to long-term brain adaptability. The model is based on three core pillars: (1) Nature and Nurture, emphasizing the interaction between genetic predispositions and environmental influences; (2) Structural-Functional Neurocognition, focusing on neuroplasticity, brain reserve, and compensatory scaffolding; and (3) Motor Behavior, which explores the role of executive functions, representations, and autonomy in skill acquisition and learning efficiency. A key aspect of SMART COMPASS is emphasizing physical fitness and autonomy-supportive environments to promote cognitive-motor performance. For example, in aging populations, SMART COMPASS can guide tailored interventions combining cardiovascular training with task-specific motor learning to maintain executive function and reduce fall risk. Similarly, structured motor programs supporting autonomy and self-efficacy can enhance motor competence and academic performance in child development. It highlights how exercise, self-efficacy, and autonomy-supportive environments can enhance neuroplasticity and learning potential, providing practical insights for motor skill development, rehabilitation, and lifelong cognitive-motor optimization interventions.

## 1 Introduction

**FIGURE 1:**
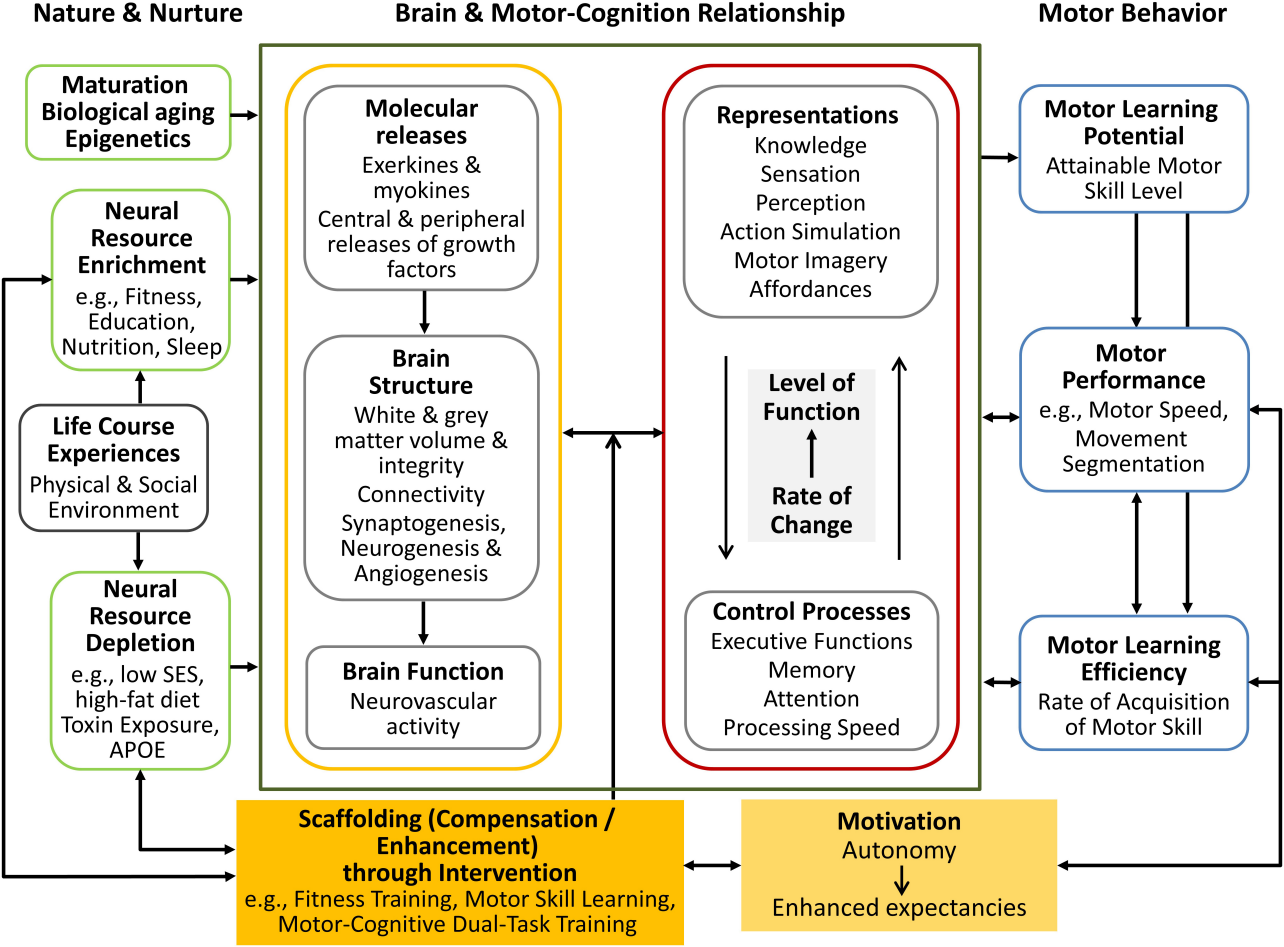
The adapted SMART COMPASS model illustrates the interplay between nature and nurture, highlighting both positive and negative influences on neurocognitive and motor performance. This version emphasizes the dynamic relationship between structural and functional brain processes, cognitive mechanisms, and motor learning, as well as the role of compensation and enhancement through intervention and motivation in shaping neural and cognitive performance, ultimately impacting motor behavior. This is a modified version of the figure published in Schott and Klotzbier (2018).

The Scaffolding Theory of Maturation, Cognition, Motor Performance, and Motor Skill Acquisition (SMART COMPASS) provides a comprehensive framework for understanding the dynamic interplay between motor and cognitive processes throughout the lifespan. This integrative model, first introduced by Schott and Klotzbier (2018), builds upon established theories to elucidate the interconnections between cognition, motor performance, and the various factors influencing their development over time (Reuter-Lorenz and Park, 2014, 2024; Ren et al., 2013). The SMART COMPASS model addresses the intricate relationship between the brain and motor and cognitive systems by offering a structured approach to assess and predict human motor performance from childhood through late adulthood. In doing so, it responds to limitations of previous models, which often focused on isolated aspects – such as neural structure, cognitive function, or motivation – without fully capturing their interaction across different life stages. By integrating these domains, SMART COMPASS provides a unified and developmentally sensitive perspective (Figure 1).

The framework is grounded in three key theoretical foundations. The first is the “Scaffolding Theory of Aging,” proposed by Reuter-Lorenz and Park (2014, 2024), which describes how the brain compensates for age-related decline by recruiting additional neural resources and how lifestyle factors (e.g., physical activity and cognitive stimulation) can support this adaptive process. The second is the “Integrated Framework for Cognitive and Motor Skill Development” by Ren et al. (2013), which emphasizes the close relationship between cognitive functions and motor performance, and illustrates how these domains develop in parallel – exerting reciprocal influence, especially during periods of rapid growth or age-related decline. The third is the “OPTIMAL (Optimizing Performance Through Intrinsic Motivation and Attention for Learning) Theory” by Wulf and Lewthwaite (2016), which emphasizes the critical role of autonomy and self-efficacy expectations as key motivational factors in enhancing motor performance and learning.

By synthesizing these theoretical perspectives, the SMART COMPASS model offers a comprehensive framework that captures the dynamic interplay between structural brain plasticity, behavioral learning processes, and motivational factors. It emphasizes the continuous, bidirectional interaction between cognitive and motor systems, highlighting how these components adapt to one another across the lifespan. Rather than adhering solely to traditional approaches prioritizing abstract thought or neural mechanisms, the model expands its scope by recognizing the essential influence of motor behavior and the environment on cognitive processes. This perspective aligns with the principles of “Embodied Cognition,” as articulated by Wilson (2002) and Foglia and Wilson (2013), emphasizing that cognitive functions are fundamentally grounded in sensorimotor interactions with the surrounding environment.

In addition, the SMART COMPASS model aligns with evidence-based research highlighting the significance of environment, motivation, and skill development in promoting lifelong motor and cognitive health. Combining these elements makes it a versatile tool for designing interventions and predicting developmental trajectories in cognitive and motor domains.

## 2 First pillar: nature and nurture

### 2.1 Genetic predispositions and environmental influences

The exploration of factors influencing human behavior often revolves around two contrasting scientific perspectives. On the one hand, some approaches underscore the pivotal role of genetic predispositions, suggesting that innate biological traits predominantly govern behavior. On the other hand, opposing views emphasize the primacy of environmental factors, positing that individual behavior is primarily shaped by external conditions and life experiences (Georgiades et al., 2017; Rangaswami, 2021). Rather than adhering to these polarized perspectives, the SMART COMPASS model establishes this dynamic, reciprocal interplay as its foundational pillar (see Diamond, 2009). This perspective emphasizes the interaction between genetic traits and experiences in shaping human behavior (Diamond, 2009; Lange and Schwarz, 2015).

Thus, integrating nature and nurture offers a comprehensive framework for understanding behavior across the lifespan (see McAuley et al., 2021, discussing the impact of genetic and environmental factors on athlete development; Zi, 2025, regarding genetic and environmental influences on motor milestones). While some of these factors are genetically determined [e.g., apolipoprotein E (APOE)], others, such as physical activity, nutrition, or intellectual engagement, can be modified to promote brain health and mitigate cognitive decline across the lifespan (Reuter-Lorenz and Park, 2014; Phillips, 2017). For example, the ε4 variant of apolipoprotein E (APOE) has been identified as a significant genetic risk factor for Alzheimer’s disease (AD) and a wide range of other neurodegenerative conditions (Windham and Cohen, 2024). Even in such cases of genetic predisposition, early identification of risk factors like APOE ε4 can inform timely and targeted preventive interventions to preserve cognitive performance. The model and the following description place particular emphasis on the role of environmental factors within this interplay, as these factors can be actively shaped. It highlights how life course experiences accumulated over time have the potential to either enhance or diminish neural resources, thus impacting cognitive and motor development. Life course experiences refer to the age-related sequence of roles, opportunities, and constraints that is an individual’s experience across the lifespan; for example, research examines how young people choose personal experiences, interpersonal relationships, and social settings in ways that reflect their past and contribute to their future (Johnson et al., 2011). This foundational understanding sets the stage for subsequent components of the SMART COMPASS model, offering a holistic view of the intricate connections between genetics, environment, neural mechanisms, cognition, and motor behavior.

### 2.2 Neural resource enrichment and depletion

Various factors throughout the human lifespan shape the enrichment and decline of neural resources (Clemenson et al., 2015). Enrichment occurs through influences that enhance brain structure and function, particularly during childhood and adolescence when the brain exhibits high plasticity (Miguel et al., 2019). During this phase, stimulating environments rich in sensory inputs and opportunities for exploration play a crucial role in optimizing cognitive development. Positive early experiences, such as social interactions, access to educational opportunities, sleep, nutrition, and exercise, are vital for promoting neural development and preparing the brain for future challenges (Schoentgen et al., 2020; Tooley et al., 2021; Schott et al., 2023; Bosseler et al., 2024). Although the brain’s capacity to adapt decreases with age, neuroplasticity persists throughout life (Power and Schlaggar, 2017). Intellectual engagement, physical exercise, and maintaining social connections positively influence cognitive performance in middle and late adulthood (Herzog et al., 2009; Mora, 2013; Reuter-Lorenz and Park, 2024). Furthermore, factors such as higher education levels (Milgram et al., 2006), physical fitness, and bilingualism or multilingualism have been associated with delayed cognitive decline and a reduced risk of dementia (Bialystok et al., 2007; Alladi et al., 2013; Anderson et al., 2020). Mishra and Gazzaley (2014) and Kolb and Muhammad (2014) highlight the potential of targeted cognitive training and enriching life experiences to enhance neuroplasticity, strengthen neural and cognitive functions, and build resilience across the lifespan.

Depletion of neural resources arises from factors that negatively impact brain structure and function. Prenatal factors, including maternal smoking, low birth weight, and stress, as well as postnatal exposure to environmental toxins, can significantly impair motor and cognitive development (Lin et al., 2017; Puga et al., 2024; Nivins et al., 2023; Nolvi et al., 2023). Additional factors, such as poor nutrition, exposure to toxins, or lack of sensory and emotional stimulation, can hinder brain development (Garner et al., 2012; Cusick et al., 2021; Pizzol et al., 2021; Cardenas-Iniguez et al., 2022; Sheridan et al., 2022). In adulthood, lifestyle choices such as smoking, high-fat diet consumption, and sedentary behavior contribute to vascular risks, diabetes, brain atrophy, and cognitive dysfunction (Debette et al., 2011; Lane et al., 2020; Zou et al., 2024). Head trauma, chronic stress, and depression further exacerbate the depletion of neural resources (Lupien et al., 2018).

## 3 Second pillar: structural and functional neurocognition

### 3.1 Structural and functional brain changes and developmental trajectories of cognitive domains

The SMART COMPASS model highlights the interaction between the brain’s structure, function, and the dynamic processes that support cognitive abilities throughout the lifespan. Significant structural changes occur during early childhood, with primary sensory and motor areas maturing early to enable fundamental abilities such as seeing, hearing, and moving (Huang et al., 2015). In contrast, more complex regions, such as the prefrontal cortex, develop later (Tsujimoto, 2008), undergoing a phase of synaptic overproduction (“wild growth”), followed by pruning. This process strengthens frequently used neural connections while eliminating unused ones, creating a highly efficient and adaptable brain (Sakai, 2020). In later adulthood, structural changes include cortical thinning, reduced white matter, and decreased synaptic connectivity (Fjell and Walhovd, 2010; Hedman et al., 2012). These changes manifest functionally as reduced neural interactions, dysregulated resting states (Persson et al., 2007), and a loss of specialization, especially in the prefrontal cortex (Park et al., 2004; Koen and Rugg, 2019).

The model indicates that life experiences, motor learning, motor skill learning, motor-cognitive dual-task training, and fitness training significantly affect brain structure and function through complex biological mechanisms (Domingos et al., 2021; see also Solis-Urra et al., 2024). Environmental enrichment (e.g., physical activity, intellectual engagement, and social interactions) triggers the release of exerkines and myokines, such as lactate and irisin, along with cytokines like interleukin-6, from muscle and fat tissue. Additionally, growth factors such as brain-derived neurotrophic factor (BDNF), vascular endothelial growth factor (VEGF), and insulin-like growth factor (IGF) are released in the central and peripheral nervous systems. These molecular processes drive neurogenesis, supporting the formation of new neurons and synaptogenesis, facilitating new neural connections. VEGF further promotes angiogenesis, the development of new blood vessels, enhancing cerebral blood flow and oxygen delivery. These structural changes help preserve and enhance the integrity of gray and white matter, maintaining brain adaptability and resilience [an overview of the possible mechanisms is described in Tyndall et al. (2018) and Dupuy et al. (2024)]. On a functional level, these structural adaptations improve neurovascular activity (Tarumi et al., 2025), connectivity within neural networks (Moore et al., 2022), and Default Network Modulation (Reuter-Lorenz and Park, 2024), leading to measurable enhancements in cognitive performance. This is particularly evident in areas such as working memory, cognitive flexibility, and inhibition (Voss et al., 2020; Moore et al., 2022).

These adaptations are not merely biological changes but actively support higher-order cognitive functions such as working memory, attentional control, cognitive flexibility, and inhibition. Aerobic and resistance training, for instance, have been shown to enhance working memory and inhibitory control in older adults (Voelcker-Rehage and Niemann, 2013). They also improve the efficiency of motor representations by strengthening neural networks involved in movement planning, execution, and sensorimotor integration (Goble et al., 2009; Taubert et al., 2011). Increased connectivity between prefrontal, parietal, and motor areas – especially in older adults – supports complex motor coordination and reflects enhanced neural integration (Heuninckx et al., 2008; Ward and Frackowiak, 2003). Such functional connectivity improvements facilitate better top-down control and coordination between executive and motor systems, which is particularly relevant in cognitively demanding motor tasks like dual-task performance (Seidler et al., 2010; Li et al., 2001; Voelcker-Rehage and Niemann, 2013). Together, these findings highlight the essential role of structural and functional plasticity – shaped by molecular processes, life experiences, and physical activity – in preserving and enhancing cognitive-motor performance throughout the lifespan.

### 3.2 Development of representations and control processes

Rather than treating cognition as a single, uniform construct, the model highlights the importance of differentiating between distinct cognitive abilities – an especially relevant approach when examining motor performance and learning processes. In their review, Ren et al. (2013) distinguish between the development of (neuronal) representations – often referred to as internal models or schemas – and executive control processes (executive functions), which play a central role in our model.

The functional association between action representations, sensation, perception, feed-forward or feedback control strategies, internal models, and the ability to mentally represent actions is imperative for motor action planning and control (Wolpert et al., 1995). The integration of information regarding the position and velocity of a limb in space, based on sensory feedback and prior experiences, will enable an individual to control their movement more accurately and complete it to achieve a desired outcome. Suppose a child exhibits an inability to plan or execute motor movements effectively. In that case, this will result in a diminished capacity to establish connections between motor movements and other domains of information, such as emotions or cognitive tasks (e.g., mathematics, reading, and problem-solving). This individual will experience an alternative manner in which the body influences cognition, signifying its substantial involvement (through alterations in perception and attention, variances in behavior, or the stimulation of neural motor systems). This entails a disparate impact of embodiment (Eigsti, 2013). It is well established that children exhibit less coordinated movement than adolescents and young adults. Children with motor and/or cognitive impairments [e.g., developmental coordination disorder (DCD), Autism, and Down syndrome] demonstrate increased temporal and spatial variability, reduced anticipatory adjustments, and variations in their capacity to generate and detect information about affordances and invariant structures (Adams et al., 2014; Schott, 2025). As the body and brain undergo a series of physical, physiological, and cognitive changes during childhood and adolescence, it stands to reason that the representation of the self and, by extension, the representation of action and its affordances also change with age (Schott, 2025). Motor imagery (MI) is a widely used experimental paradigm for studying cognitive aspects of action planning, control, and embodied cognition. According to Decety and Grèzes (1999), this phenomenon is characterized as an active cognitive process whereby the representation of a particular action is internally reproduced in working memory, devoid of any overt motor output from a first-person perspective. Many studies have indicated that MI processes are likely present in early childhood. Evidence of this can be seen in the speed-accuracy trade-off in imagined movements (e.g., hand laterality judgment paradigm) of children aged approximately 7 years. However, this relationship is more evident in older children. As children mature, the durations of imagined movement approach actual execution durations (for an overview, see Schott, 2025). Concurrently, the accuracy of MI improves gradually during development (Toussaint et al., 2013). Specifically, 11-year-olds demonstrate significantly higher MI accuracy levels than 7- and 8-year-olds (Toussaint et al., 2013), suggesting that action simulation processes undergo continuous refinement during childhood.

Representations also mirror crystallized intelligence, drawing on accumulated knowledge and experience. In contrast, fluid intelligence, involving problem-solving and logical reasoning, is closely connected to executive functions, as it supports the creation of new representations and promotes adaptive thinking. These cognitive abilities follow different developmental trajectories. Fluid intelligence typically peaks in young adulthood and then gradually declines, whereas crystallized intelligence remains relatively stable or can increase through ongoing knowledge acquisition (Salthouse, 2004; Lindenberger and von Oertzen, 2006; Craik and Bialystok, 2006).

Representations become more complex and precise over time (Strick et al., 2021). Singleton et al. (2021) showed that motor representations in the motor cortex of rats become more complex and precise as they develop, undergo reorganization, and can be modified explicitly through motor learning. Representations include declarative knowledge, such as facts and concepts, and procedural skills, like motor skills. These structures are stored in specialized neural networks in the premotor and parietal cortex and are adapted through repeated use, experience, or motor learning (Seitz et al., 2008). Although the formation of representations slows in early to middle adulthood, most knowledge systems stay robust into late adulthood as long as they are regularly utilized (Watrin et al., 2022).

Executive functions – a common division of executive functions is into cognitive flexibility, inhibitory control, and working memory (Miyake et al., 2000) – likewise follow distinct development patterns. Inhibition and cognitive flexibility emerge early in childhood, although the latter continues to mature into adolescence (Clark et al., 2013). Working memory shows substantial improvements from childhood (Broomell and Bell, 2022; Moriguchi et al., 2016) through adolescence and refines further during early adulthood (Ferguson et al., 2021). Later in life, declines in processing speed, working memory capacity, and cognitive flexibility often lead to slower reaction times and reduced multitasking abilities (Idowu and Szameitat, 2023). Age-related declines in executive functions can make retrieving stored representations more challenging or their formation less efficient, even if they remain intact at the neural level (Shadmehr and Holcomb, 1997; Pedraza et al., 2024).

Crucially, the interaction of representations and executive functions underpins learning and adaptability. While executive processes facilitate the establishment, organization, and use of new knowledge structures, representations stay more resilient when regularly accessed and practiced. This reciprocal relationship underscores the importance of continuous cognitive and physical engagement in meeting evolving life demands and preserving cognitive performance over the long term.

## 4 Third pillar: motor behavior

### 4.1 Impact of cognition on motor behavior across the lifespan

According to the previous description of the model, developmental processes, maturation, and biological aging processes lead to neuronal changes that ultimately influence cognitive abilities. While representations are largely preserved throughout the lifespan, executive functions experience a notable decline in older adulthood. This chapter highlights the intricate interplay between cognitive performance and motor domains, including motor learning potential, performance, and efficiency.

Motor learning potential refers to the individual’s capacity and readiness to acquire, refine, and adapt motor skills in contexts that offer learning opportunities through practice and experience. It has been hypothesized that it may mediate the relationship between static neurocognition and functional outcome (Budoff and Friedman, 1964). Motor performance describes the execution and quality of motor tasks, reflecting skill performance in specific activities. Motor learning efficiency demonstrates the speed and effectiveness of acquiring and retaining motor skills, often influenced by cognitive strategies (implicit or explicit), practice conditions, and feedback mechanisms. In older adulthood, motor learning remains feasible but occurs more slowly and requires greater effort, reflecting a reduction in efficiency (Rodrigue et al., 2005; Voelcker-Rehage, 2008; Yan et al., 2010; Ehsani et al., 2015).

**FIGURE 2:**
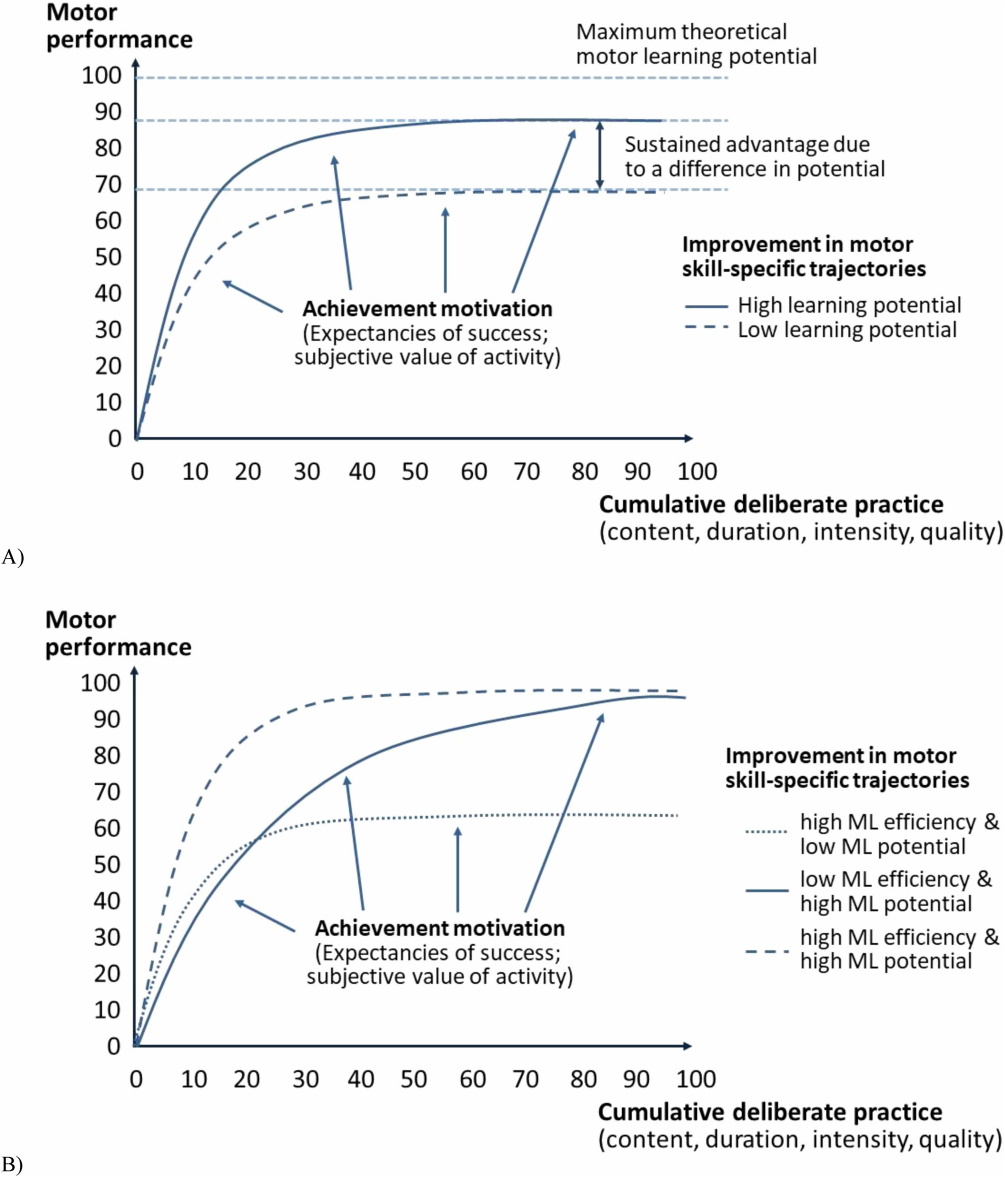
Efficiency and potential of motor performance development trajectories, considering achievement motivation and deliberate practice. **(A)** Advantages based on differences in improvement potential (low vs. high learning potential). **(B)** Differences based on motor learning efficiency (ML, low vs. high) and improvement potential (low vs. high learning potential).

Figure 2A on the progression of motor skill development shows that two individuals learn at the same rate but approach different motor skill levels. Depending on various factors (e.g., achievement motivation and deliberate practice), neither individual may reach the maximum theoretical potential of the motor skill-specific level. However, the individual who develops motor skills toward a higher potential will gain an advantage that a competing individual will never achieve. The main difference between differences in learning rate and differences in potential is that some individuals must overcome additional hurdles to eliminate skill advantages. The advantages based on differences in learning rate are eliminated by continuing to gain experience in deliberate practice. Individuals must change their training activities to eliminate advantages based on differences in motor learning potential rather than repeating them. Because the required changes may be difficult to observe and offer uncertain benefits (e.g., risks or trade-offs), differences in potential add an additional level of difficulty to an otherwise simple task (see also Challenge Point Framework; Guadagnoli and Lee, 2004). The skill development pathways in Figure 2B illustrate how different combinations of motor learning rate and skill development potential can lead to different individual advantages. An individual whose motor skills develop toward a high potential at a high learning rate will enjoy a temporary advantage over individuals who improve toward a high potential at a lower learning rate, and a permanent advantage over individuals who develop their motor skills at a higher learning rate.

The motor learning potential is particularly high during early developmental phases, with critical periods playing a vital role in shaping motor skills. Experiences during these phases have lasting effects, while insufficient motor stimulation can lead to persistent deficits. Although motor learning potential diminishes with age, adults can still develop or restructure motor skills, albeit more slowly and with increased effort (Etnier et al., 2001; Rodrigue et al., 2005; Voelcker-Rehage, 2008). This demonstrates the enduring plasticity of the brain, even in later life. Motor performance follows a clear trajectory: steady improvement from childhood to early adulthood, followed by a gradual decline in later life (see Holfelder and Schott, 2022 for object control skills; Marchesi et al., 2022 for balance-related motor performance). Upper limb motor behavior also shows age-related changes, particularly in tasks involving fine motor control such as writing, grasping, or manipulating small objects. These tasks depend heavily on cognitive resources, especially working memory and executive functions. Consequently, individuals with reduced cognitive capacities – due to developmental disorders or age-related decline – often exhibit lower fine motor performance (Corti et al., 2017; Dahdal et al., 2016; Liou et al., 2020). The relationship between motor skills and cognitive abilities is particularly evident in fine motor tasks (Hoogendam et al., 2014; Corti et al., 2017), with stronger correlations indicating that lower cognitive functioning is typically accompanied by poorer fine motor performance. Stronger correlations are observed in individuals with developmental disorders (Dahdal et al., 2016) or age-related cognitive decline (Liou et al., 2020), but also for object control skills (Holfelder and Schott, 2024). Age-related degeneration in the prefrontal cortex, which is responsible for storing and retrieving motor representations, also impacts motor performance (Seidler et al., 2010).

Based on the work of Ren et al. (2013), the SMART COMPASS model highlights the crucial interplay between knowledge structures (representations) and executive functions in linking cognitive and motor functions. It emphasizes the dynamic and reciprocal relationship between cognitive and motor systems across the lifespan. Motor representations, or mental movement models, are generally acquired through repeated practice and are mainly executed automatically, especially for familiar and well-learned movements. Their basic execution does not depend heavily on executive functions (Yogev-Seligmann et al., 2008). Moreover, motor behavior can also reinforce cognitive functions. For instance, engaging in complex motor activities may stimulate executive control, planning, and attentional resources, particularly in early developmental phases and later adulthood (Voelcker-Rehage and Niemann, 2013; Diamond, 2000). This reciprocal dynamic supports the notion that structured motor skill training can serve as a valuable tool for cognitive enhancement.

Motor representations, or mental models of movement, remain relatively stable, ensuring continuity in motor performance even in older age. In contrast, executive functions, including working memory and cognitive flexibility, decline significantly with age, primarily affecting motor representations’ formation, adaptation, and strategic retrieval when required in novel or complex situations (Kennedy et al., 2008). This decline and age-related degeneration in the prefrontal cortex – a key region for managing executive functions and motor representations – impact cognitive and motor performance.

## 5 Scaffolding as a unifying concept of adaptive brain strategies: integrating brain reserve, cognitive reserve, maintenance, compensation, and enhancement

*Brain Reserve*, *Cognitive Reserve*, *Brain Maintenance*, *Compensation*, and *Cognitive Enhancement* are central concepts that describe the brain’s resilience to aging and pathological processes. They address distinct mechanisms while complementing each other by highlighting various aspects of the brain’s structural and functional adaptability (Stern, 2009; Stern et al., 2019, 2020, 2023; Pettigrew and Soldan, 2019; Kremen et al., 2022; Cabeza et al., 2018; see Box 1).

**BOX 1 Key concepts.**

*Brain Reserve* refers to the structural capacity of the brain, such as synaptic density, cortical thickness, or the volume of gray matter. This passive capacity enables the brain to tolerate neuronal damage before clinical symptoms emerge (Stern et al., 2020). It is influenced by genetic factors and shaped by lifelong education and activity, which can enhance reserve over time (Nyberg et al., 2012).

*Cognitive Reserve* focuses on the functional flexibility and adaptability of the brain. This reserve allows for reorganizing neural networks or alternative strategies to maintain cognitive functions despite structural damage (Stern, 2009). It represents a dynamic mechanism shaped by intellectual, social, and occupational experiences (Cabeza et al., 2018).

*Brain Maintenance* aims to preserve the integrity of neural structures and functions. Preventive measures, such as regular physical activity, a balanced diet, and cognitive stimulation, play a crucial role by reducing age-related changes and pathological processes, such as atrophy or amyloid plaque formation (Nyberg et al., 2012; Marchesi et al., 2022).

*Compensation* describes short-term, reactive mechanisms that address performance deficits. In such cases, the brain recruits additional or alternative networks to accomplish specific tasks and maintain cognitive or motor performance (Reuter-Lorenz and Park, 2014; Cabeza et al., 2018).

*Cognitive Enhancement* (Hildt and Franke, 2013) refers to strategies to improve cognitive abilities such as attention, memory, or problem-solving in healthy individuals. Dresler et al. (2018) categorize these strategies into three dimensions: biochemical (e.g., caffeine and modafinil), physical (e.g., TMS and tDCS), and behavioral (e.g., cognitive training, meditation, and physical activity). Each approach differs in mechanisms, effectiveness, and side effects. The authors highlight the need for a nuanced understanding of these distinct enhancement forms.

These concepts are not confined to late adulthood or pathological decline but are integral throughout the lifespan (Lin et al., 2023; Reuter-Lorenz, 2002). Brain Reserve contributes to early developmental processes by supporting the maturation of brain structures, facilitating performance enhancement through enriched experiences (de Rooij, 2022; Morris et al., 2021). Cognitive Reserve plays a pivotal role during childhood and adolescence, aiding in developing efficient cognitive strategies and bolstering superior cognitive performance via learning and skill acquisition (Schoentgen et al., 2020; de Rooij, 2022). Brain Maintenance is crucial in preventing age-related decline and earlier life stages, where it supports optimal development and sustained neural functioning, promoting cognitive enhancement and lifelong brain health (Lin et al., 2023). Compensatory processes are active across the lifespan, enabling individuals – regardless of age – to adapt dynamically to novel or complex cognitive demands, thus maintaining performance levels through various challenges (Reuter-Lorenz, 2002). Cognitive Enhancement is not limited to adulthood but plays a role across the entire lifespan, supporting cognitive performance optimization from early development through old age. From childhood onward, enhancement strategies – such as cognitive training, physical activity, or enriched environments – can strengthen neural efficiency and promote long-term brain health and adaptability (Dresler et al., 2018; Hildt and Franke, 2013).

### 5.1 Scaffolding: a unifying concept

The concept of scaffolding integrates and extends these mechanisms by emphasizing the active construction, adaptation, and strengthening of neural “Scaffolds” that support both structural and functional changes (compensation vs. enhancement). Scaffolding is a dynamic process incorporating Brain Reserve, Cognitive Reserve, Maintenance, Compensation, and Enhancement elements. In the present article, we approach scaffolding as a comprehensive, lifespan-encompassing mechanism that bridges these distinct concepts and highlights the interplay between structural integrity and functional adaptability. Focusing on the active and ongoing processes of neural reorganization, we emphasize how (developmental) scaffolding contributes to resilience and adaptability across the lifespan and under varying conditions of challenge or pathology. Crucially, we emphasize not only protective and compensatory effects in aging but also the constructive, performance-enhancing potential of scaffolding in early and middle life stages. This includes childhood, adolescence, and young adulthood, where scaffolding supports development and growth under conditions of increased cognitive demand or opportunity (Cabeza et al., 2018; Carranza-Pinedo and Diprossimo, 2025; Reuter-Lorenz and Park, 2014). In relation to Brain Reserve, it contributes to structural resilience by building new neural connections and stabilizing existing ones. This enhances structural capacity and actively supports long-term stability. In early development, scaffolding fosters the establishment and refinement of neural circuits, thereby boosting reserve and enabling higher cognitive capacities (Nyberg et al., 2012; Stern et al., 2020). Like the concept of Cognitive Reserve, scaffolding endorses using alternative networks and strategies. However, it distinguishes itself by focusing on the active development and restructuring of neural connectivity rather than relying solely on previously accumulated experiences (Cabeza et al., 2018). This is particularly relevant in periods of developmental plasticity, where scaffolding helps build and refine neural systems that underpin emerging cognitive abilities.

Additionally, scaffolding complements the goals of Brain Maintenance by promoting the stabilization and preservation of neural networks. This preventive component helps delay age-related degeneration and strengthens structures essential for cognitive functions (Nyberg et al., 2012). This process delays age-related degeneration in older adults and supports the continuous strengthening and optimization of brain function during development and adulthood. From childhood through late life, scaffolding mechanisms help preserve and enhance the efficiency of neural processing, whether by preventing decline or by supporting peak performance in learning, creativity, and problem-solving (Nyberg et al., 2012). In Compensation, scaffolding is key in improving preparedness for functional limitations. Constructing neural scaffolds establishes a foundation for effective compensatory mechanisms, thereby maintaining motor and cognitive performance (Reuter-Lorenz and Park, 2014, 2024). Beyond compensatory functions, scaffolding also enhances cognitive and behavioral flexibility throughout the lifespan. In youth, it facilitates the acquisition of new skills; in adulthood, it supports career and social adaptability; and in older age, it sustains performance despite neurobiological change. Scaffolding is thus an all-encompassing mechanism that unites aspects of Brain Reserve, Cognitive Reserve, Maintenance, Compensation, and Cognitive Enhancement without being confined to any single concept. It reflects both short-term adaptations and long-term development, acting as a core process of neurocognitive resilience and growth across the lifespan. Building functional and structural scaffolds allows the brain to develop and sustain resilience against age- and disease-related changes, while also enabling enhancement, optimization, and growth in earlier life stages. This concept of scaffolding offers a detailed perspective on the complexity of neural adaptation processes throughout the lifespan (Cabeza et al., 2018; Stern et al., 2020; Nyberg et al., 2012).

#### 5.1.1 Scaffolding through fitness training

Dasso (2018) differentiates between physical activity, exercise, and physical fitness. The relationship between these concepts shows that physical fitness can be understood as a measurable outcome and goal achieved through regular physical activity and targeted exercise. Physical activity includes all bodily movements, while exercise aims explicitly to improve fitness (health-related and skill-related). We emphasize distinguishing these terms, particularly in scientific and clinical contexts, as they have distinct meanings and applications (Dasso, 2018; Corbin et al., 2000; see Box 2). In the SMART COMPASS model context, we understand fitness training as a deliberately planned, structured, and regularly performed exercise to improve or maintain physical fitness. The objective is, on the one hand, to induce specific adaptations in neuronal structure and function through targeted training stimuli, and on the other hand, to enhance motor performance.

**BOX 2 Distinguishing physical activity, exercise, and fitness.**

*Physical activity* is the broadest category, encompassing all forms of movement (Dasso, 2018) and refers to any bodily movement produced by skeletal muscles that increases energy expenditure above resting levels, regardless of whether it is planned, structured, or repetitive. Examples include walking, gardening, or climbing stairs.

*Exercise*, on the other hand, is a specific subset of physical activity. It is deliberately planned, structured, and performed regularly to improve or maintain physical fitness or health. Examples include jogging, swimming, or strength training. In the Medical Subject Headings (MeSH) system, exercise is described as a form of physical activity that is generally structured, consistent, and undertaken to enhance or preserve physical health and fitness (National Library of Medicine, 2025; Centers for Disease Control Prevention (CDC), 2017; World Health Organization (WHO), 2018).

*Physical fitness* describes the state of physical health and performance achieved through regular physical activity and healthy lifestyle habits. Caspersen et al. (1985) define physical fitness as “the ability to carry out daily tasks with vigor and alertness, without undue fatigue, and with ample energy to enjoy leisure-time pursuits and to meet unforeseen emergencies.” This definition has evolved over time to describe a combination of attributes related to an individual’s capacity to perform physical activity. Physical fitness is now understood as a multidimensional concept comprising two primary components: health-related aspects, such as cardiovascular fitness, muscular endurance, strength, flexibility, and body composition, and skill-related aspects, including agility, balance, coordination, reaction time, and speed (Corbin, 1991). This includes measurable attributes such as cardiovascular fitness, muscular strength, flexibility, and balance, which can be assessed using VO_2_ max or strength tests (Corbin et al., 2000).

Evidence from scientific studies underscores the critical role of physical activity and exercise in promoting neural adaptations and cognitive benefits, with impacts observed in children, adolescents, and older adults. In *childhood*, regular physical activity has been consistently associated with improvements in executive function, working memory, attention, and academic performance. Studies by Erickson et al. (2015) and Chaddock-Heyman et al. (2015) show that children with higher levels of aerobic fitness demonstrate better cognitive flexibility and learning outcomes. Structural and functional brain adaptations underpin these behavioral effects. Neuroimaging research reveals that greater physical activity in children is linked to increased hippocampal and basal ganglia volumes, improved white matter integrity, and more efficient neural processing (Chaddock-Heyman et al., 2014). Khan and Hillman (2014) emphasize that physical activity during early developmental stages supports hippocampal and prefrontal cortex development, enhancing memory, attentional control, and higher-order cognition. The underlying neurobiological mechanisms include increased levels of BDNF, improved synaptic plasticity, and enhanced efficiency of neural circuitry. Importantly, intervention studies confirm a dose–response relationship, with higher physical activity levels yielding stronger cognitive benefits. These findings are reinforced by meta-analytic data: a comprehensive umbrella review and meta-meta-analysis by Ludyga et al. (2023) demonstrates that regular physical activity – particularly aerobic training – significantly improves executive functions and memory performance in children (see also Meijer et al., 2020). During *adolescence*, a similarly positive influence of physical activity is observed, particularly as this developmental stage involves substantial structural and functional brain reorganization. Belcher et al. (2021) report that physical activity during adolescence contributes meaningfully to emotional regulation, cognitive control, and psychological resilience. Herting and Chu (2017) likewise find that higher aerobic fitness correlates with better academic performance and cognitive functioning in adolescents. Structural changes in the adolescent brain mirror these behavioral improvements. Exercise promotes the maturation of the prefrontal cortex and hippocampus – regions essential for executive functioning and self-regulation – while also supporting increases in gray matter volume, greater neural connectivity, and the refinement of brain networks. These findings suggest that adolescence is a critical window for promoting cognitive and emotional resilience through regular physical activity.

In *adulthood*, the benefits of physical activity continue (Zhang et al., 2023). Adults who engage in regular exercise demonstrate enhanced attention, working memory, cognitive flexibility, and processing speed. The 2018 *Physical Activity Guidelines Advisory Committee Scientific Report* provides robust evidence supporting the role of physical activity in maintaining and improving various executive functions (see Hillman et al., 2008). These behavioral outcomes are supported by neurophysiological adaptations, such as enhanced neurovascular function, increased neurogenesis, and improved integration of functional brain networks. Such changes support cognitive performance in the short term and preserve brain health over the long term, serving as a buffer against age-related cognitive decline. The importance of physical activity becomes even more pronounced in *older adulthood*, where it functions as a protective factor against cognitive deterioration, dementia, and functional impairment. Erickson et al. (2015) found that physically active older adults experience significantly slower memory and attention declines than their sedentary peers. At the structural level, regular exercise mitigates brain atrophy, particularly in the hippocampus and prefrontal cortex, and enhances functional connectivity between neural regions. Umegaki et al. (2021) further highlight that exercise elicits adaptive changes across structural, physiological, and molecular domains, including upregulation of BDNF, improved cerebral blood flow, and enhanced network integration. These adaptations contribute to what Liu-Ambrose et al. (2018) describe as “Neural Scaffolding” – a system of reinforced cognitive resilience that helps counteract the impact of aging, neurodegenerative diseases (Brown et al., 2013), and traumatic brain injury (Archer, 2012; Zhang et al., 2022).

Across all life stages, the effects of physical activity on the brain and cognition are driven by both acute and chronic mechanisms. On an acute level, physical activity temporarily increases neurotransmitter availability and cerebral perfusion, which results in short-term enhancements in cognitive performance, such as improved attention and mental clarity. In the long term, chronic physical activity fosters more profound neurobiological changes, including heightened neurogenesis, stronger synaptic connections, and enhanced neural network efficiency. Aerobic fitness is a critical mediating factor in these processes, which supports cardiovascular health and facilitates the delivery of oxygen and nutrients to the brain – an essential foundation for neuroplasticity and cognitive maintenance. These findings are substantiated by a pivotal study by Dupuy et al. (2024), published in *Nature Human Behaviour*. In their work, the authors challenge the conclusions of Ciria et al. (2023), who had argued against a causal link between physical activity and cognitive enhancement. Dupuy et al. (2024) present compelling evidence that physical activity yields meaningful cognitive benefits throughout the lifespan. These effects are mediated by several mechanisms, including increased secretion of neurotrophic growth factors like BDNF and VEGF, structural brain changes such as increases in white and gray matter volumes, and functional improvements such as enhanced synaptic plasticity and neuronal connectivity. The study also highlights the moderating effects of exercise type, intensity, duration, age, and gender on these outcomes. Together, these findings provide strong empirical support for global public health recommendations – such as those from the World Health Organization – which advocate for regular physical activity not only as a means of physical wellbeing but also as a vital strategy for preserving and enhancing cognitive function, promoting brain health, and building lifelong psychological resilience.

#### 5.1.2 Scaffolding through motor learning

Although Park and Reuter-Lorenz (2009) do not explicitly address motor learning, they suggest that activities promoting cognitive and physical engagement can enhance neurocognitive plasticity and adaptability in older adults. In the early stages of learning, broad neural networks are initially activated to serve as scaffolds for acquiring new motor skills. These networks are particularly engaged when tasks are novel and demanding. However, with continued practice, this broad activation is gradually replaced by more specialized and efficient neural pathways (Bassett et al., 2011). A similar pattern was observed by Bassett and Mattar (2017). As learning progresses, the brain shifts from widely connected structures to a more optimized and focused organization. As sensorimotor systems gain autonomy, reliance on cognitive control processes decreases. This suggests that practice refines broad neural networks into specialized and efficient structures, thereby enhancing both performance and efficiency in specific tasks (Bassett et al., 2015).

The concept of scaffolding describes this process of initially widespread neural activation, which establishes a foundational structure for acquiring new skills (Petersen et al., 1998; Park and Reuter-Lorenz, 2009). This mechanism is relatively inefficient in early learning phases and requires significant cognitive and motor effort. However, with continued practice, activity shifts to specialized and efficiently connected networks, enabling the smooth and automated execution of learned motor skills. Makino et al. (2016) add that during early learning phases, new dendritic connections are formed and stabilized, while redundant connections are pruned with continued practice, increasing the efficiency and stability of the network. Notably, initially formed scaffolds – such as motor-cognitive representations or compensatory neural networks – may become underutilized over time but can be re-engaged and strengthened later in life in response to new challenges, such as age-related decline or novel task demands. This re-engagement reflects the brain’s capacity for use-dependent plasticity and supports the notion that scaffolds can be maintained in a latent state and recruited as a functional reserve when needed (Park and Reuter-Lorenz, 2009; Lövdén et al., 2013). Motor learning relies on the dynamic interaction of various neuronal adaptation processes, involving specific brain regions and mechanisms. Studies in mice (Hwang et al., 2022) indicate that motor learning triggers specific neuronal changes in the motor cortex and related circuits. Hwang et al. (2022) showed that motor learning activates motor engram neurons in the primary motor cortex (M1), correlating with performance. This process involves dendritic remodeling, stabilization of new synapses, and strengthened corticostriatal connections, enabling long-term storage of motor representations. Chen et al. (2015) highlighted changes in inhibitory (GABAergic) circuits, with reduced activity, facilitating dendritic spine reorganization critical for learning. Qiao et al. (2022) demonstrated that newly formed dendritic spines are task-specific, becoming more active during performance, thus enhancing task specificity, retaining motor skills, and allowing adaptation to new tasks without interfering with previously learned skills. Collectively, these findings underline the role of structural and functional plasticity in motor learning.

The study by Spampinato and Celnik (2021) outlines how error-based learning, reinforcement learning, use-dependent learning, and strategy-based learning have distinct neurophysiological foundations and functions. *Error-based learning* relies on sensory prediction errors to calibrate internal models for movement adjustment. The cerebellum plays a key role in this process, processing errors through Purkinje cell activity and promoting long-term depression at synapses. A reduction in cerebellar inhibition has been observed during this process, indicating increased connectivity between the cerebellum and the primary motor cortex (M1). *Reinforcement learning*, on the other hand, is driven by the reward of successful movements, leading to long-term potentiation-like changes in M1, which stabilize motor skills. The basal ganglia are central to this process, as they process reward prediction errors and modulate M1 plasticity through dopaminergic signaling. *Use-dependent learning*, triggered by repeated movements, reduces variability and reorganizes cortical maps in M1. This repetition fosters synaptic changes and the formation of new dendritic spines via Hebbian plasticity. *Strategy-based learning* complements these mechanisms by utilizing cognitive processes like planning and problem-solving, with the dorsolateral prefrontal cortex playing a key role.

In early learning phases, often referred to as the “cognitive phase” (Fitts and Posner, 1967), but also for complex motor tasks, the dorsolateral prefrontal cortex activity is closely linked to developing and applying these strategies. The study by Spampinato and Celnik (2021) emphasizes the importance of these process shifts during learning. Error-based learning, driven by the cerebellum, dominates in the early stages, while reinforcement and use-dependent mechanisms, rooted in M1 and the basal ganglia, become more prominent later. Strategy-based learning is important in the early stages when addressing cognitively demanding tasks that involve the prefrontal cortex.

These findings highlight that motor learning is driven by a flexible and dynamic interplay of neuronal processes, providing valuable insights for developing targeted scaffolds. Such scaffolds serve as compensatory mechanisms to support motor recovery in neurological conditions like stroke or traumatic brain injury and play a crucial role in enhancing cognitive and motor functions across the lifespan, including in healthy aging.

### 5.2 Supporting autonomy and addressing self-efficacy expectations

The SMART COMPASS model highlights that a crucial factor in promoting participation in physical activity or structured fitness training is the consideration of psychological needs and creating conditions that enhance autonomy and self-efficacy (Wulf and Lewthwaite, 2016). In this context, the OPTIMAL theory of motor learning, developed by Wulf and Lewthwaite (2016), describes fundamental mechanisms that take these factors into account and facilitate both motor skill acquisition and performance improvement (see also Wulf et al., 2010). Their theory is based on three principles: autonomy, enhanced expectations, and an external focus of attention, with autonomy and expectancies central to the SMART COMPASS model.

Autonomy refers to the perception of control over one’s learning process. Wulf and Lewthwaite (2016) demonstrated that even small choices, such as deciding the order of exercises or the type of feedback received, can significantly improve motor learning. Autonomy support increases intrinsic motivation, reduces stress, and strengthens self-efficacy. When learners feel a sense of control over their performance, they engage in deeper processing of motor information, leading to improved movement quality and long-term retention of skills. Wulf and Lewthwaite (2016) understand that self-efficacy expectancy refers to a person’s belief in their ability to perform a specific task successfully. This concept is based on Albert Bandura’s notion of self-efficacy but is applied in her work within the context of motor control and motor learning. Wulf and Lewthwaite (2016) emphasize that motor skills can be optimized through physical practice and by strengthening individuals’ belief in their abilities. Their research shows that individuals with confidence in their abilities perform better in motor tasks. This effect can be achieved through positive feedback, social comparisons, or structuring tasks to ensure frequent success experiences. Their research thus highlights the close connection between psychological concepts like self-efficacy, motor learning, and athletic performance.

These elements shape motivation and cognitive engagement during learning, promoting more effective skill development and long-term retention (Wulf et al., 2014; Bacelar et al., 2024). The theory emphasizes the interaction between autonomy support and enhanced expectancies, highlighting how these factors reinforce each other in the learning process. When individuals have a sense of control over their learning, they are more likely to believe in their success, boosting their motivation and strengthening their autonomy (Ghorbani and Bund, 2020). From a practical perspective, coaches, teachers, and trainers should actively support autonomy by offering choices in the learning process and fostering a supportive environment. At the same time, they should enhance learners’ expectations by providing positive and constructive feedback while ensuring they experience frequent successes. This combination improves movement quality and increases motivation and overall enjoyment of the learning experience (Lemos et al., 2017; Ghorbani, 2019).

By incorporating this perspective into the SMART COMPASS model, it becomes evident that motor learning extends beyond mechanical repetition. Psychological factors, such as autonomy and positive performance expectations, are crucial in optimizing skill acquisition and retention. When these principles are applied, educators and trainers can cultivate more effective and engaging learning environments, ultimately leading to improved motor skill development and long-term success.

## 6 Limitations and future directions

While many of the components integrated into SMART COMPASS, such as scaffolding, environmental factors, structural and functional brain changes, neuroplasticity, or executive functions, are well supported by empirical research, the model as a unified framework has not yet been systematically tested. Its multidimensional structure poses challenges for empirical operationalization, particularly when accounting for interindividual differences and contextual variability (e.g., cultural, socio-economic, or environmental factors). Additionally, the strength and nature of the relationships between the model’s core components, such as motor behavior, cognitive control, and motivational factors, are likely to vary across developmental stages. For instance, motor and cognitive interactions may be powerful during early childhood or older adulthood, while other phases may involve different patterns or degrees of integration. These developmental and contextual differences should be further examined to refine the model and strengthen its generalizability and translational value. To enhance the empirical utility of the SMART COMPASS model, future research should aim to operationalize its key mechanisms through well-defined experimental paradigms. This includes testing how structural and functional scaffolds are formed through specific training protocols and how motivational variables such as autonomy and self-efficacy interact with neuroplastic mechanisms. Future research should focus particularly on the neural mechanisms of scaffolding in motor learning and fitness training. It is crucial to investigate how scaffolding emerges in different learning phases and to identify methods that specifically activate neural networks, as well as training approaches that contribute to the stable formation of neural connections and functional scaffolds (Spampinato and Celnik, 2021; Hwang et al., 2022; Qiao et al., 2022). Another important research area concerns the moderating effects of autonomy and self-efficacy expectations (Wulf and Lewthwaite, 2016; Ghorbani and Bund, 2020). Autonomy in training design and positive performance expectations can enhance motivation and positively influence neuroplastic adaptations and the structural integrity of the brain (Lemos et al., 2017; Erickson et al., 2015). In this context, the interaction of dopaminergic and cholinergic systems could play a particularly crucial role (Umegaki et al., 2021; Solis-Urra et al., 2024).

The SMART COMPASS model provides valuable insights into the targeted enhancement of motor and cognitive performance through scaffolding. Future research should focus on exploring the underlying neural mechanisms and developing optimal intervention strategies to strengthen brain integrity (Dupuy et al., 2024; Domingos et al., 2021). Integrating motivational factors such as autonomy and self-efficacy could further promote long-term cognitive and motor improvements (Wulf et al., 2014; Bacelar et al., 2024).

## 7 Conclusion

The SMART COMPASS model provides an integrative framework for explaining motor and cognitive performance as well as learning processes across the lifespan (Schott, 2019; Schott and Klotzbier, 2018; Reuter-Lorenz and Park, 2024). It describes individual differences in motor learning potential, efficiency, and performance by combining neurobiological, cognitive, and motor perspectives (Ren et al., 2013). Additionally, it enables predictions regarding motor performance and learning efficiency while facilitating the targeted development of interventions aimed at sustainably optimizing and stabilizing motor and cognitive performance.

The model explains how functional and structural adaptations in the brain (second pillar), shaped by experiences and environmental factors (first pillar), serve as the foundation for cognitive processes and, in turn, influence motor behavior (third pillar). These adaptations involve changes in neural plasticity, synaptic connectivity, and the structural integrity of brain areas involved in cognitive and motor processes. Through targeted training or learning experiences, new neural connections can form, or existing networks can be utilized more efficiently (Park and Reuter-Lorenz, 2009; Bassett et al., 2011). The model describes this process as scaffolding, a dynamic adaptation mechanism in which the brain constructs temporary neural structures to acquire and stabilize (or maintain) new cognitive and motor skills. These neural scaffolds initially support the processing and storage of new information, become more efficiently utilized with continued practice, and are eventually transformed into permanent, specialized networks (Makino et al., 2016; Bassett and Mattar, 2017). These networks serve as a foundation for optimized cognitive and motor performance and act as compensatory mechanisms and reserves that help counteract age-related decline or neurological impairments (Cabeza et al., 2018; Stern et al., 2020; Reuter-Lorenz and Park, 2014). By developing alternative processing strategies and mobilizing unused resources, the brain can compensate for performance losses and maintain functionality despite structural changes (Stern et al., 2023; Nyberg et al., 2012). In addition, cognitive enhancement strategies – such as targeted training or stimulation – can actively promote the formation and strengthening of these neural scaffolds, accelerating their efficiency and long-term integration into functional networks (Dresler et al., 2018).

## Data Availability

The original contributions presented in this study are included in this article/supplementary material, further inquiries can be directed to the corresponding author.
